# Microstructure Semiconductor Materials and Optoelectronic Applications

**DOI:** 10.3390/nano16050336

**Published:** 2026-03-09

**Authors:** Zhou Wang, Xiaoyan Liu

**Affiliations:** College of Integrated Circuit Science and Engineering, Nanjing University of Posts and Telecommunications, Nanjing 210023, China; zhouwang@njupt.edu.cn

## 1. Introduction

As information technology advances relentlessly toward higher speed, lower power consumption and higher integration, conventional bulk materials and planar device structures are confronting a series of fundamental limitations. Consequently, achieving highly efficient optoelectronic signal conversion at reduced scales and lower power consumption has emerged as a critical challenge. In response, extensive and in-depth explorations have been conducted within the field of semiconductor optoelectronics, striving to surpass the performance boundaries of traditional bulk materials and planar devices. Within this context, microstructure semiconductor materials and devices, owing to their distinct advantages in harnessing light-matter interactions, have proven to be a pivotal pathway for realizing high-performance optoelectronic applications [1,2,3].

Diverse strategies have been employed to engineer high-performance semiconductor materials that enable precise control over photon and carrier behavior. The foundational band engineering of microstructure semiconductor materials necessitates addressing the challenge of multi-dimensional and synergistic modulation within the material system. Thus, selecting semiconductor material systems with an appropriate bandgap, high carrier mobility, and excellent thermal stability is crucial for developing optoelectronic devices with both high performance and integrability [4,5]. The integration of low dimensional semiconductor systems including quantum wells, quantum dots, and nanowires with photonic crystal structures is regarded as a significant breakthrough in optoelectronic integration. This combination not only broadens the spectral response range of the materials, but also potentially facilitates efficient carrier injection and extraction at heterointerfaces through quantum confinement effects [6,7,8,9]. These unique characteristics lay the physical groundwork for developing more efficient optoelectronic conversion devices, which can be broadly applied in cutting-edge information technology scenarios [10,11,12].

Furthermore, to meet the increasingly urgent demands for low power consumption and high integration of optoelectronic applications, relevant studies have explored various technical routes, achieving multi-functional optoelectronic fusion on a single chip through heterogeneous integration of semiconductor materials with different bandgaps and dimensionalities. By taking advantage of the regularity brought about by bandgap regulation and specific device structures, the quantum efficiency, modulation speed and spectral selectivity of semiconductor devices have been significantly enhanced [13,14,15,16].

## 2. An Overview of Published Articles

Hu et al. [17] proposed a chemically specific, label-free nanophotonic biosensor for distinguishing multiple analytes. The sensor used a simple resonant metasurface composed of a metal-insulator-metal (MIM) structure, with the insulating layer made of dynamic material Ge_2_Sb_2_Te_5_ (GST). The device achieved ultra-broadband resonance coverage across the 5–10 µm range by tuning the crystallization degree of the GST dielectric spacer layer. They further demonstrated that excellent fingerprint reconstruction was achieved when the vibrational fingerprint aligns with the resonant wavelength.

Kang et al. [18] worked on a top-gate coplanar amorphous indium–tin–gallium–zinc oxide (a-ITGZO) thin-film transistors (TFTs) with a split channel structure fabricated on SiO_2_/p-type Si substrates. They investigated the effect of pulse rising time on AC stress-induced performance degradation in the devices through experiments and simulations. Using the stretched exponential model, they attributed the threshold voltage shift degradation of the devices to both electron trapping in the bulk states of the gate dielectric and trapping at the channel-dielectric interface traps.

Shen et al. [19] systematically reviewed the current state of research and future challenges related to interface thermal resistance in heterostructures within micro-nano power devices. They analyzed heat transfer mechanisms at typical heterointerfaces and novel low-dimensional materials and discussed the limitations and applications of the current interface thermal resistance characterization techniques. They also proposed new ideas to overcoming the thermal management bottlenecks of micro-nano power devices.

Wang et al. [20] proposed a high-bandwidth underwater optical communication (UWOC) system which utilized micro-light-emitting-diodes (micro-LEDs) with Non-Return-to-Zero On-Off Keying (NRZ-OOK) modulation. Through simulations, they significantly enhanced the optoelectronic performance of micro-LED by dimensional scaling and quantum well layer reduction. They successfully implemented underwater audio communication at an 11.5 m UWOC distance using an ultra-low incoming optical power level (12.5 μW) at the photodetector site, which provided valuable experience for optimizing UWOC systems.

Chen et al. [21] constructed a flexible vacuum-ultraviolet (VUV) photodetector based on Al nanoparticles (Al NPs) surface-enhanced boron nitride nanosheets (BNNSs). They found that the introduction of Al NPs significantly enhanced BNNSs light absorption at 185 nm via LSPR, and improved carrier transport through interface barrier modulation, thereby increased the photocurrent. They revealed the coordination physical mechanism of plasmonic enhancement, providing a reference design for flexible VUV photodetectors.

## 3. Conclusions

In summary, this Special Issue focuses on the latest research progress in the device design, device fabrication, and optoelectronic applications of microstructure semiconductor materials. Addressing themes related to high performance optoelectronic devices, researchers have conducted systematic explorations into photodetectors, micro-light-emitting devices, optical modulators, transistors, and power devices based on microstructured systems such as quantum wells, quantum dots, nanowires, and two-dimensional material heterojunctions.

The potential solutions explored in this Special Issue for critical challenges including higher integration, higher optoelectronic conversion efficiency, lower power consumption and higher reliability, which are poised to have a profound and positive impact on light emission, detection, data transmission, intelligent sensing, and energy management in both current and next-generation information societies.
